# Current News and Opinion

**Published:** 1886-04

**Authors:** 


### THE AMERICAN DENTAL ASSOCIATION.

### WHERE SHALL THE NEXT MEETING BE HELD, CHICAGO OR SAN FRANCISCO ?

At a meeting in Buffalo, in December, at which six members of the Executive Committee were present, an informal meeting was held and a vote was taken, resulting in five for Chicago, one for Buffalo. It was the understanding after the vote that the final decision and completion of arrangements should be left with the Committee of Arrangements, they to be governed by circumstances in the final decision. Since more recently, a proposition to hold it in San Francisco has been made, it is deemed best to submit the question to the profession.

The reasons for going to California have already been so fully given in an article in the dental journals, and more recently in a circular letter, that it is unnecessary for me to take space to present them again. They read well, and make us all feel like saying Go! *Why, of course!* But facts are hard things to knock against, and it will be found much easier for the majority of the dental profession to say to some other fellow, "Go!" than it will be to muster the money and spare the time to go himself. The rank and file of our profession are not rich men. The question of expense has to be considered by many.

A large per cent. are young men that need the meeting, and the Association needs them. How many will feel that they can spare the time and money necessary for so long a trip ? Calculate a week to go, a week to return, the best of a week for the meeting, and two weeks to see California and the Great West, including points of interest on the route, and five weeks are gone. Fare from Chicago, round trip, $62.50. Meals and sleeper, about $5 per day. Expenses, $5 per day at lowest estimate, at a time when the city and surrounding country are thronged with the Grand Army and thousands of strangers. Add to this, extra railroad and steamboat fare for all side trips to points of interest, and the extras that you can never plan for, and it is easy to see by all who have traveled much that $300 would be a *very* low estimate for the expense, besides at least five weeks of time. Those who have been to California all coincide in the statement that one cannot see enough of California and the West to pay them for going without a greater expenditure of time and money than we have named. It is well known that July and August are the most disagreeable months in which to make the trip to California, and we see the country at its worst. These are the months when Californians get away if they can. Since learning the desire of some that the American Dental Association should be held in San Francisco, the California Dental Societies have very courteously invited us, and we are sure their hospitality will be fully appreciated by all, but there are times when we cannot afford to accept even hospitality. This is one. The Association would lose too much. We could not hope for any large accession of new members, nor for the new ones gained to often meet with us from so great a distance, and we should lose many.

Chicago was selected on account of its being central, easily accessible from all parts of the country at low railroad rates, and of its having unsurpassed hotel accommodations, and cool summers. The fact that a hundred of the new members elected last year were Western men and should be held, also entered into the decision. Your Committee have been quietly planning and working since the last meeting to insure at our next the largest attendance and most successful meeting ever yet held, feeling that each meeting should be an advance upon the one that precedes in points of numbers and interest; that we ought not only to hold the new members gained last year, but that we should add as many more at our coming session. It is too soon to complete definite railroad arrangements, but if the decision is for Chicago, we expect to bring them within the reach of all.

At a meeting of the Chicago members of the American Dental Association and of the Chicago Dental Society, called to ascertain the views of the profession here, March 17th, the following resolution was adopted by a vote of twenty-seven to five:

"*Resolved*, That it is the sense and desire of this body that the next meeting of the American Dental Association should be held in Chicago, and that we extend to the Association a most cordial and hearty invitation to meet with us."

AN EXCURSION TO CALIFORNIA AFTER THE MEETING.

Your Committee are assured by the railroad authorities that they can have equally as favorable rates in all respects for an excursion upon close of the meeting, if any considerable number wish to go to California, as are promised for the Association. The Committee will see that *no pains are spared* to make such an excursion a success, if enough signify their wish to go to warrant making the arrangements. Thus, none who wish to go will be deprived of seeing California at the reduced rates, while great numbers will not be deprived of attending the Association because they cannot afford a trip to California. It is a serious question whether the Society has a right to hold its meetings beyond the reach of so large a class. Let us remember that our Association is a scientific body, intended to reach and benefit *the great body of the profession* as far as possible.

There has been so much discussion of the whole subject, in the Committee and out, and the Committee are at such distances from each other, that it is impossible to get a united expression of views *in time for the journals*, as promised, hence I submit this as an expression of a part of the Committee. Am very sure that each member of the Committee wishes to do only the very best thing for the Association, and to carry out the wishes of its members, and your votes will show us what those are, and greatly facilitate the work of the Committee.

Please answer *promptly* by letter or postal the following questions:

In your judgment, should the meeting be held in Chicago or San Francisco?

Do you expect to attend the meeting if held in San Francisco?

Do you expect to attend the meeting if held in Chicago?

If the meeting is held in Chicago, will you probably go to California after the meeting is over, if an excursion is decided upon?

J. N. Crouse,

Chairman Ex. Com. and Com. of Arr.

2101 Michigan Ave., Chicago, Ill.

TO THE MEMBERS OF THE AMERICAN DENTAL ASSO'N.

AND OTHER DENTISTS.

SALEM, MASS., March 20, 1886.

DEAR DOCTOR :—Desiring that you should have all the facts in my possession, bearing upon the question of a selection of time and place for the next meeting of the American Dental Association, I again address you upon the subject. Since the organization of the Executive Committee last year, there has never been a formal meeting of the committee. At the time of what is known as the Buffalo Conference, last November, it was found that a majority of the Executive Committee were present, and so an informal meeting of those present was held and the matter of a place for our next meeting was discussed. The Secretary of the Committee stated that he had received urgent official letters of invitation for the Association to meet in Buffalo, from the Buffalo Dental Association and the 7th and 8th District Societies of New York. The Chairman objected to Buffalo, stating that in his opinion there was not sufficient hotel accommodations, and said that Chicago was the only proper place to hold the meeting. To this one member said he had spoken to a number of our leading members from Chicago, and they had expressed themselves in opposition to our meeting there next time. To this the Chairman replied that if the Committee would put their feet down upon the idea of accepting any kind of a reception or banquet, a thing, which in his opinion, we ought now to do, there would be no objection to our meeting in Chicago. After some further discussion an *informal* vote was taken; votes being cast for Buffalo, St. Louis and Chicago, though the latter had the greatest number. It was then agreed that the members of the Committee should ascertain all they could about railroad rates to and from various places that would be suitable for the meeting and correspond or have some future meeting of the Committee for decision. Such is the record. After I had received the assurance, from agents of the transcontinental lines, that my efforts to get the Grand Army rates to San Francisco would probably meet with success, being in New York at the meeting of the Odontological Society, where, among a large body of dentists present, were many of our members, I began to tell them what rates we could have if we wished to have the meeting in San Francisco. I found there was much enthusiasm upon the subject, and many at once declared they would go. "Well, we ain't going all the same," exclaimed the Chairman, who was present arguing against the idea of going. I simply replied that I thought we should if the members wished to do so. Dr. C. then began complaining of me for talking about the matter at all, saying, "just hold up now, and let the Committee get together and decide this thing." He was told by myself and one other of the Committee present that the matter was of sufficient importance to submit to the members for their judgment, and that there was then an opportunity to consult with nearly fifty of our members. There the matter dropped. I had previously written to several of the Executive Committee, and all from whom I had heard replied strongly favoring the idea of going. The Chairman did not answer my letter. I had made a formal application to the Transcontinental Pool for the Grand Army rates and privileges for us if we wished to avail ourselves of them. That was all. The pool disbanded without reaching action on my application.

I then enlisted the services of a friend, Past Commander-in-chief Vanderwort, of the G. A. R., who is Special Agent of the Union Pacific Railway, in our behalf, and through him the rate and privilege was granted us provided we would hold our meeting the last week in July, and which would certainly be the best time as you will see later on. Enclosed is the guarantee the railroads made, and the Southern Pacific and Northern Pacific have endorsed it.

My previous circular gave you information with regard to rates from other points. The rate has not been definitely fixed east of points represented by St. Paul, Chicago, St. Louis, New Orleans, etc., beyond the fact that they will not exceed half fare. When I sent you the last circular, it was suggested to me by all of the Committee of Arrangements except the Chairman, that I should send it out *officially* as Secretary; but, as what I had done had been simply as a member, with no personal end in view, I did not do so. I only sought to place information before you and was not anxious to parade my official title before you. The Chairman of the Committee hastened to inform you that it was your duty to address all correspondence on a subject he had not given you any information about, to him, as Chairman. You were requested not to "hamper the committee by giving a premature decision." As the Committee had not taken any action in the matter except that a majority of them had declared in favor of going to California, I suspect that if that postal had read, "Do not hamper the *Chairman* who desires we shall meet in Chicago," it would have stated the fact in the case a little plainer. In my former circular I stated that the California Dental Societies had invited us cordially. See accompanying circular for the several invitations.

In response to my circular of inquiry, every officer of the Association but one has replied favorably, and that one admits that "there is no doubt that we should have a large meeting." I gave you extracts from the letters of the others in my last circular. Six of the nine Executive Committee are in favor, and only one is understood to be decidedly opposed. Two of the three Committee of Arrangements are decidedly in favor. All of the Chairmen of Sections but one will be at San Francisco if we go, and that one is not quite certain yet about going, but he is decidedly opposed to Chicago. At Minneapolis half of the Chairmen of Sections were absent. Nearly one hundred members have declared in favor of California. At Minneapolis only fifty-six old members were present. About one hundred and fifty dentists, who will all be members at San Francisco, have already declared they shall be present, and every mail is swelling the number. Read what some of the members write in letters received since my last circular. Secretary Harlan writes, "I am in favor of San Francisco if the larger number desire. *I am not in favor of Chicago this year.*" Treasurer Keeley writes, "Had we known all the facts when at Minneapolis, three-fourths of the members would have voted for San Francisco. We should not lose this opportunity." Dr. Keeley is not in favor of Chicago at all. Dr. Thompson, of Executive Committee, writes, "I am opposed to Chicago, and in favor of California. Hope a good Kansas delegation will attend, as is most likely." Dr. Fredericks, of Executive Committee, writes, "If we are to be the National Dental Association of this country, we should not be confined to any part of this Union in our meetings, but must take in the whole country. I do not see why anyone should object to our meeting in California. I think it will prove a big thing for us to meet there." Dr. Darby writes, "Put me down as in favor of San Francisco. A great many dentists who are not in the habit of attending will avail themselves of this opportunity." Dr. Stockton writes, "I am in favor of San Francisco." Dr. Newby, of St. Louis, writes, "Without a single exception, every dentist with whom I have spoken has favored San Francisco." President Barrett writes, "A National Society should represent the whole nation. Our Pacific States should be brought into the American Dental Association." Dr. Bodecker writes, "Count me in for San Francisco." Drs. Butler, Bill and Horton, of Cleveland, all write, "I favor San Francisco." Dr. Noble, of Washington, writes, "The feeling this way is unanimous for San Francisco. There will be five or six new delegates from here." Dr. H. W. Morgan writes, "You may record my vote in favor of the meeting on the Pacific Coast. I cannot go myself, but I do not desire to be in the way of others who can. Feel sure you will have an interesting trip." Dr. Fuller writes, "I hope the Association will meet in California." Dr. Cook writes, "Heartily approve of holding meeting in San Francisco." Dr. Leiter, "I say Amen! to the California meeting." Dr. Mariner, "Would prefer San Francisco to any place on this continent." Dr. Gaylord, "By all means *let's go.*" Dr. Patten, "I am for San Francisco to a man." Dr. A. E. Brown, of Chicago, "The proposition fully meets with my approval" Dr. Martin, Chicago, "Would like the California meeting better than any other." Dr. Harroun, "Never banked money that paid me so good interest as my trip to California." Dr. Knepper, "Heartily in favor." Dr. Moore, Dakota, "Go! it will be a grand trip." Dr. Case, "To hold the meeting at San Francisco will be the correct thing on general principles. No doubt it will meet with the success it deserves." Dr. Field "prefers San Francisco." Dr. Holmes, "Think it due the dentists of the Pacific Coast that the American Dental Association should meet there." Dr. Doud writes, "I favor San Francisco" Dr. Brackett writes, "The plan you present has great merit, in that it would contribute to make the Association more truly a National body" Dr. Parmly Brown, "I am in favor of California if the majority wish it." Dr. Gilmer, "The selection of San Francisco would be acceptable to me." Dr. Tibbetts, "I have spoken to a number of the boys here, and they are all in favor of San Francisco." Dr. Eundonberg, "I vote for San Francisco, and my vote cannot be changed." Dr. Butler, "Am still in favor of San Francisco." Dr. Waters, "In view of the general expression I do not say nay."

Only nine or ten members have sent unfavorable responses. And so the record goes on from a much larger number of the members than the number of old members who were present at Minneapolis. Let us see what is the record from societies and States outside of the Association who will send delegates. Last year there were present from all New England only three persons. This year there will go to California not less than twenty-five, and every State in New England will send delegates. The New England Dental Association, which is next to the largest in the country, will send a large delegation. The H. Society has just elected its full quota of delegates. From eight societies in New

York come favorable reports. New Jersey is on the list. The President of the Pennsylvania Odontological and the Secretary of the State Society write earnestly in favor, and from several other societies in that State come reports of delegates. Maryland will send delegates. The District of Columbia will be well represented. Favorable letters have also come from the South, especially in South Carolina, Louisiana and Texas. Ohio, Michigan, Indiana, Illinois, Wisconsin, Minnesota, Missouri, Iowa, Kansas, Nebraska, Dakota, Colorado, all will have delegates. California and Oregon, with nearly seven hundred dentists, *will have its full quota* of delegates. The reports from these distant sections are only just beginning to come in. Every dental society in the country will be furnished with credentials, and instructed as to their right to representation in our Association. Now shall we say to our members on the Pacific Coast, who have kept up their membership and paid their dues year after year, we won't go ? To that great body of our profession on the Pacific Coast who want to unite with us, and who stand ready to receive us, as Dr. Atkinson says, "in a manner fully equal to that extended to us at London," we don't care for your membership ? Shall we deny them the privilege of greeting and joining us, and not let them have the benefit of our counsel, but repel them ? Shall we say to them "we are *not* the American Dental Association of the country; we are the American Dental Association of a particular section ? Shall those of us who possibly cannot go to California be selfish enough to prevent the meeting from being held there ? Many will not attend the meeting wherever held. It is hoped such will not prevent that, which in their judgment is for the best interests of the Association, being done.

It is proposed that we meet in Chicago. We have met there twice; and there are other cities, like St. Louis and others of the West, where we have been invited, that have a prior claim upon us. Besides, nearly one-half of our present members in Chicago have favored San Francisco, and are opposed to Chicago. The President and many members of the Illinois State Society have written earnestly favoring San Francisco. Some of the most prominent of our members from Chicago do not favor our meeting there this year. Among them may be mentioned Drs. Allport, Harlan, Brophy, Haskell, Talbot, Ames, Marshall, Griffin, and others. Dr. Allport writes: "As between San Francisco and Chicago, I am for the former. As between Chicago and 'Tophet,' I don't care, for with the row that will be kicked up, the temperature of the two places would be about the same." It seems to me that those who remember the unpleasant episode which occurred among the Chicago dentists at Minneapolis, it would be most unwise to meet in Chicago this year. If it is not best to go to California, let us go to St. Louis or some other western city, or to Buffalo, where we have been cordially invited, or some other eastern point. Dr. Crouse himself writes, under date of February 22: "Remember I have little personal preference, and that little is against having it in Chicago."

It is urged that by going far away from where we met last year we shall lose many of those we gained then. That is certainly not complimentary to those who joined us last year, and implies we must always meet near them. From none of our members have there come more urgent requests to go to California than from Minnesota, when we met, and the other western States west of Illinois. The Secretary of the St. Louis Society writes: "The members of the St. Louis Dental Society will go as a body for San Francisco." Dr. Martindale, writing for Minnesota, hopes "we shall go because it will establish the fact that we have no geographical bias." Vice-President Smith, of Minneapolis, wrote they would fill one car from there. Drs. Hunt, Kulp, Wilson, and others, from Iowa, favor California; and so on all through the west, among the very ones who joined us last year.

It is proposed to have an excursion from Chicago to California after the meeting, via the Northern Pacific. Is it likely that these members and delegates from the west who want to go to San Francisco, will come away east to Chicago first and spend a week of their time there? Is it likely that the members and delegates from the northwest are coming to Chicago first? Will those from the South, from Louisiana and Texas, and who can go to California via the Southern route, come away north to Chicago to join an excursion? Besides such a scheme would not get us to California until the last of August, when vacations usually expire and we have to hurry home. I would also call your attention to the following from the circular issued by the railroads with reference to the Grand Army rates and privileges extended to us. "Excursion tickets will not be sold to go west to San Francisco *via Portland, Oregon*, and return east via either of the other routes mentioned."

Great stress has been placed upon the matter of expense to deter you from going. This is a matter which you are all mathematicians enough to calculate for yourself. The opportunity is a great one, and a man can make the expense great or small as he pleases. The hotel prices in California are about the same as in Eastern cities, say an average of $3.50 per day, and we shall have reduced rates. If one is hurried he can make the trip in three weeks time. Four weeks or longer can be profitably employed. The last week in July will be the best time for having the meeting, because we shall then be through with our meeting and ready to participate in that grand ovation which is to be extended to the Grand Army the first week in August, and for which the State and city have secured nearly $200,000.00 with which to entertain them. It will be such an ovation and series of receptions as has never been known on this continent, and will alone be worth the trip to California. To get out to California on an excursion the latter part of August, which is not possible at the same rates and with the same privileges as earlier, would be to find the people tired out, little interested in us, and the facilities for getting about and seeing things at the low rates, not then what they will be earlier. Certainly the months of July and August are the only ones when the great body of dentists can afford to be away from their offices. It is not hot in San Francisco at that time, for the people wear the same clothing the year round, and you will need outside garments every evening.

After a careful study of the matter, and extended correspondence, covering all sections of the country, I am convinced that it would be most wise for us to hold our next meeting in San Francisco, the last week in July. I hope no one will think there are any personal differences among the majority of the officers and Executive Committee, who favor California for the next meeting, and the small minority who do not. We are all on the best of terms. Vote for any place you please. You are not obliged to choose either San Francisco or Chicago. In voting for a place you do not thereby commit yourself to going there any more than you have in past years. At the present date nearly a hundred members have indicated their preference for San Francisco, and there is no doubt but there will be as many delegates as last year if we meet there.

Fill out the enclosed blank postal card, and mail it as early as possible, that we may know the decision of the members. Address it to the one whom you think the proper officer to receive it.

Respectfully, A. M. DUDLEY.

At the last moment a telegram received from the Chairman of the Invitation Committee of the San Francisco Dental Association, informs us that the dentists of the Pacific Coast are entirely unanimous in their desire to have the next session of the Association held in San Francisco. A. M. D.

---

INVITATION OF THE CALIFORNIA DENTISTS.

At a meeting of a general committee representing every dental organization on the Pacific Coast, the following resolutions were adopted:

1st. WHEREAS, A wish or willingness has been communicated to us by the Executive Committtee of the American Dental Association, to hold its annual meeting in San Francisco, in August next, and the meeting of that body among us is calculated to increase the professional and public regard for the character and culture of our profession; and

WHEREAS, The meeting of that national body among us will be an event in the history of our profession, long sought and earnestly desired; and

WHEREAS, The consideration of our professional ideas of a modern, theoretical and practical nature, and a comparison of them with those of the past, will be elevating, interesting and beneficial to the profession, individually and collectively, as well as to the people generally; and

WHEREAS, A visit to this State of another organization, also another element of our Eastern co-laborers and fellow-citizens, will give additional opportunities for the investigation of our domestic and social institutions, our educational facilities, as well as our commercial, agricultural and mineral advantages; therefore

*Resolved*, That we cordially invite the co-operation of all dental societies or associations, both State and local, that may be entitled to representation in the national Association, as well as the general profession—the people—to join us in receiving said body in a manner in keeping with the reputation of the citizens of this coast for acts of philanthropy, public spirit and generosity.

2d. WHEREAS, Communications have been received by various members of the profession, expressing a desire of the Executive Committee of the American Dental Association to fix upon San Francisco as the place for its next annual session (August, 1886); and

WHEREAS, The occasion is opportune for such a meeting here, in consideration of the inducements offered to travel, that will prevail during the convention of the Grand Army of the Republic in August of this year; and

WHEREAS, The meeting of the American Dental Association on this coast, and if possible the National Association of Dental College Faculties and Examiners, at the same time, will do much to strengthen the bonds of profession and fellowship between us and our Eastern co-laborers, as well as to elevate the professional standard in this section; therefore be it

*Resolved*, That the societies represented by this Committee, viz.: The California State Odontological Society, the Southern California Odontological Society, the Faculty of the Dental Department of the University of California, and the Alumni Association of the same Department, do cordially invite the American Dental Association to hold its next meeting in San Francisco.

Therefore, in accordance with the above facts, I am instructed by the said Committee to extend a most cordial invitation to the American Dental Association to hold its annual session of 1886 in this city, August next.

Hoping for a favorable response at the earliest practicable moment, I am

Very truly yours, H. J. PLOMTEAUX, Sec'y,
General Committee on Invitation and Arrangements, 531 Sutter Street.

---

### AN INVITATION FROM CHICAGO DENTISTS.

At a meeting of the Chicago members of the American Dental Association and of the Chicago Dental Society, called to ascertain the views of the profession, March 17th, the following resolution was adopted by a vote of twenty-seven to five :

*Resolved*, That it is the sense and desire of this body that the next meeting of the American Dental Association should be held in Chicago, and that we extend to the Association a most cordial and hearty invitation to meet with us.

---

### UNION AND CENTRAL PACIFIC RAILWAYS.

The Union and Central Pacific Railways have guaranteed to Dr. Dudley, for the American Dental Association, its members, delegates and all dentists and their families desiring to attend the meeting, a rate of fifty dollars for round trip tickets from the Missouri River to San Francisco and return, going by the above route and returning by any other.

The Grand Army of the Republic have selected August 3d, 1886, at San Francisco, as a proper time to hold their Encampment. Also, the Woman's Relief Corps and Army of the Potomac. The Knights Templar, after careful consideration, selected August, 1886, as a proper time for holding their Conclave at San Francisco.

August is the fruit and flower season, and the time when the Pacific Coast is at its best, and the occasion is one that will never be duplicated in this country. Should the Association conclude to go to San Francisco to hold their Convention of 1886, we can assure all concerned that the railway lines west of the Missouri River will do everything in their power to make their trip and visit to the Pacific Coast as pleasant as it can be made.

The Dental Association, if numbers enough go, will be run through on special train, such stops being made as will give them the best views of the country through which they travel, and accompanied by employees of our road, who will do everything to assist in making their trip pleasant.

J. W. Morse,

Gen. Pass. Agt.,

Omaha, Neb.

M. T. Dennis,

New England Agt., Boston, Mass.

### MISSOURI DENTAL COLLEGE.

The twentieth annual commencement of the Missouri Dental College was held, in connection with that of the St. Louis Medical College, at Memorial Hall, St. Louis, Mo., on Thursday evening, March 4, 1886.

The address to the graduates was delivered by H. H. Mudd, M. D., the valedictory by Prof. W. L. Fischel.

The number of matriculants for the session was twenty-eight.

The degree of D. D. S. was conferred by the dean, Prof. Henry H. Mudd, M., D., upon the following graduates :

Henry Louis McKellops, Missouri.

Charles Summa, Missouri.

Thomas E. Heatherly, Illinois.

William Way Hart, Illinois.

Alexander S. Holstead, Illinois.

George Larkin Mock, Missouri.

Reinhart Rembe, Missouri.

Arthur Jay McDonald, Missouri.

S. H. Fuller, Secretary of Faculty.

### NEW YORK COLLEGE OF DENTISTRY.

The twentieth commencement exercises of the New York College of Dentistry were held in Chickering Hall. Professor F. Leroy Satterlee announced that during the year 179 students had been connected with the College M. McN. Walsh, president of the Board of Trustees, conferred the degrees, and the prizes were awarded. The first prize, a gold medal, was given to John I. Hart. The second, a silver medal, was awarded to Charles H. Bush. Alfred Berghammer and Furman Clayton received the third and fourth prizes respectively. The valedictory address was delivered by Edmund E. Minner, and the address to the graduates by F. F. Van Derveer. The following are the graduates:

Atkinson, C. B.
Bornschein, E.
Berghammer, A.
Betting, H. N.
Bowman, S. J.
Bush, C. A.
Brenan, W. J.
Barr, V. G.
Burt, E.
Clayton, F.
Clegg, J. C.
Coon, W. W.
Coen, G. D.
Crandall, A. McC.
Chamberlain, F. C.
Davis, E. C.
Engel, Lewis
Frost, E. D.
Heath, W. F.
Hart, J. I.
Henry, C. De W.
Heinsdmann, F., Jr.
Jenkins, E. P.
Kenzel, W. H., Jr.
Keppy, F. B.
Koch, George
Knapp, I. W.
Lynch, J. C.
Minner, E. E.
Maynard, F. J.
Manville, G. P.
Myrick, F. A.
Onderdonk, T. W.
Palmer, B. B.
Slack, W. M.
Smith, S. E. E. C. K.
Schreiber, G. J.
Sanders, G. C.
Somers, L.
Sandhusen, G.
Stuart, L. E
Siqveland, P. S. F. M.
Schell, F. M.
Sturridge, E.
Stewart, R.
Taylor, A. Jr.,
Treadwell, T. C.
Vogt, E. S.
Vinson, J. S.
Whaley, J. C.

### KANSAS CITY DENTAL COLLEGE.

The seventeenth annual commencement exercises of the Kansas City Medical and Dental College were held at Music Hall, in that place, Tuesday evening March 16th, 1886. The list of matriculants and graduates in the Dental Department is as follows:

MATRICULANTS.

S. S. Noble, Montrose, Penna.

J. W. Heckler, Scio, Ohio.

G. W. Earle, Tacoma, W. T.

J. J. Austumell, Herman, Mo.

W. C. Love, Newton, Ks.

J. N. Chipley, Colorado

W. H. Rosenberg, California

W. F. Woodry, Solomon, Ks.

W. A. McCarter, Warsaw, Ind.

E. S. Sweet, Springfield, Mo.

R. V. Anderson, Kansas City, Mo.

W. H. Radford, Kansas City, Mo.

J. H. Parsons, Evanston, Ill.

M. Tullis, Great Bend, Ks.

Robert Lawrence, Ks.

GRADUATES.

J. N. Chipley, Colorado
M. Tullis, Kansas.

J. D. Patterson, Secretary.

---

### BALTIMORE COLLEGE OF DENTAL SURGERY.

The forty-sixth annual commencement was held Saturday, March 6, 1886, at the Academy of Music, Baltimore, Md.

The number of matriculants for the session was 102, the number of graduates was 43.

The following is a list of those graduating:

Ashbrook, Abraham L.
Baker, Geo. W., Jr.
Baynes, Harry F.
Burghard, August
Bradsher, Charles W.
Brown, Walter F.
Bryant, Emory A.
Cappel, Currey
Carroll, Howard H.
Colardeau, Chas. E.
Colding, Henry S., M. E.
Covode, R. G.
Downey, Wallace M.
Dulaney, Winfield B.
Farley, Jas. P.
Foley, J. William,
Gregg, J. F.
Hendrickson, J. Eayre
Hill, E.
Hobson, Bolling
Hubbell, Silas,
Houston, S. H., A. B.
Johnson, H. Herbert
Joyner, Henry A.
Keller, Geo. Charles
Laubach, Chas. C.
Lindsley, A. C.
Marshall, Robt. H.
Mitchell, John W.
Orrison, J. Edgar
Ovenshire, Jas. M.
Payne, L. Ernest
Pearson, Albert A.
Patterson, J. F.
Pinney, Worthington
Russell, Robert S.
St. Clair, Robert Owen
Starr, Robt. W.
Stiff, Frank W.
Summerlin, Algarine T.
VanKirk, Thos. Campbell
Weaver, Wm. H., A. B.
Wright, W. Budington.

---

### CENTRAL DENTAL ASSOCIATION OF NORTHERN NEW JERSEY.

At the annual meeting of the Central Dental Association of Northern New Jersey, Feb. 15, 1886, the following officers were elected:

*President*—Dr. B. F. Luckey, Paterson.

*Vice-President*—Dr. Geo. E. Adams, South Orange.

*Secretary*—Dr. James G. Palmer, New Brunswick.

*Treasurer*—Dr. Chas. A. Meeker, Newark.

EXECUTIVE COMMITTEE.

Dr. S. C. G. Watkins, Montclair, Dr. W. P. Richards, Orange,
Dr. Harvey Iredell, New Brunswick, Dr. Oscar Adelberg, Elizabeth,
Dr. C. F. Holbrook, Newark.

Beginning with the month of April, the meetings will be held regularly in Newark.

This society was organized in February, 1880, and is entering upon its sixth year, in a healthy and flourishing condition. It is the only local society of dentists in the State, and numbers among its members the leading gentlemen of the profession. Heretofore its meetings have been held from office to office, as has been the rule in other societies. It has recently been decided to make Newark the place of meeting, as being the most central location, and the Board of Trade rooms, corner of Market and Broad streets, have been secured for the regular monthly meeting. The evening of meeting is the third Monday of each month, except July and August. We are always glad to see members of the profession from other States, and hope that this article coming to the notice of those in our own State, who are not members, will lead them to attend our meetings and see for themselves. JAMES G. PALMER, Secretary.

---

### VERMONT STATE DENTAL SOCIETY.

The tenth annual meeting was held at Bellows Falls, commencing March 17th, and continuing three days. The following is a list of officers for the ensuing year:

*President*—J. P. Parker, Bellows Falls.
*First Vice-President*—E. E. McGovern, Vergennes.
*Second Vice-President*—W. H. Spencer, Poultney.
*Secretary*—Thos. Maund, Rutland.
*Treasurer*—James Lewis, Burlington.
*Executive Committee*—W. S. Curtis, E. S. Tracy, C. G. Campbell.

Next meeting to be held in Burlington, the third Wednesday in March, 1887.

THOS. MAUND, Secretary.

---

### DENTAL SOCIETY OF THE STATE OF NEW YORK.

The above society will hold its 18th annual meeting at Albany, Wednesday and Thursday, May 12th and 13th. The sessions will begin at ten o'clock of the first day.

The Board of Censors will meet at the same place, Tuesday, May 11th. Candidates for examination for the diploma of the society and the degree of M. D. S., may obtain full information in regard to subjects and conditions of Dr. Frank French, Secretary of the Board, Rochester. J. EDW. LINE, *Rec. Sec.*

---

### FIFTH DISTRICT DENTAL SOCIETY.

The Fifth District Dental Society of the State of New York will hold its eighteenth annual meeting at Rome, N. Y., Tuesday and Wednesday, April 13th and 14th, 1886.

Members of the profession from other societies are invited to attend and take part in the discussions. C. J. PETERS, D. D. S., Secretary.

### KANSAS STATE DENTAL ASSOCIATION.

The fifteenth annual meeting of theKansas State Dental Association will convene at Topeka, on Tuesday, May 4th, continuing three days. This meeting will be made the most interesting and profitable one in the history of the Association. Members of the profession in other States are cordially invited to be present. Topeka is easy of access, and has excellent hotel accommodations.

C. B. Reid, Secretary, Topeka, Kan.

### ILLINOIS STATE DENTAL SOCIETY.

The twenty-second annual meeting will be held at Rock Island, Ill., beginning Tuesday, May 11, 1886, and continuing four days.

Dentists in this and adjoining States are cordially invited to attend.

J. W. Wassall, Secretary,
208 Dearborn Ave., Chicago.

### A GENERAL SLAUGHTER.

Nineteen persons out of twenty have crowded teeth at the age of twelve, thirteen or fourteen. I do not mean irregularity, but such a condition as would impede a thread or quill toothpick if the attempt is made to pass it between the teeth from crown-top to gum. Such a condition is a frightful source of decay. Contact is bad, but pressure is fatal.

If all children and young people had healthy habits of diet and employment, the conditions named would hardly count. We must take these conditions as we find them, and obviate their bad consequences if possible.

The process is very simple. Extract four teeth at about the thirteenth year. If all the teeth are sound, select the bicuspids or the first molars. Choose between sound and decayed or imperfect teeth, and select the latter. Make a space thus of the width of one tooth at about the location of the second bicuspid in each jaw. This space will be filled in two or three years by all the teeth moving towards it. Thus you give freedom to the incisors. Room, boys; room for all the teeth.

This brief article, which has been quoted by some journals, is more completely crowded with false teaching than ever was a mouth with teeth. That extraction may sometimes be demanded when, through inheritance or imperfect development, there is not room for the teeth, is patent to even the blindest dental mole, but to teach that teeth should be extracted from every child indiscriminately is doctrine that can come from no one who entertains more than one idea at a time. Mastication can never be perfectly performed upon isolated teeth, and proper mastication of the food is the peculiar office of those organs. If the Creator had made man after the image of some of our dental impracticables, what a creature he would have constructed.

### THE DENTAL SECTION OF THE INTERNATIONAL MEDICAL CONGRESS.

The following resolution was adopted at the January meeting of the Chicago Dental Society, and the corresponding secretary instructed to transmit a copy to The Independent Practitioner for publication:

"*Resolved*, That this Society endorses the action of the conference at Buffalo regarding the International Medical Congress. The action is embodied in the following resolution:

" '*Resolved*, That we as members of the dental profession deem it inexpedient to recommend the organization of a Section of Dental and Oral Surgery in the International Medical Congress of 1887, under the present circumstances.' "

P. J. KESTER, Corresponding Secretary.

---

### DENTAL JOURNALS WANTED.

Cash will be paid for the following numbers of dental journals, or an exchange will be made with those who desire to complete their own files:

THE DENTAL REGISTER:

Vols. III. and VI., complete.

Vol. XXXVI., No. 1.

AMERICAN JOURNAL OF DENTAL SCIENCE (Third Series):

Vol. II., Nos. 3, 4, 5, 6, 7, 8, 10.

" V., Nos. 3, 7, 11.

" VI., Nos. 2, 3, 5, 7, 10.

" VII , Nos. 2, 3, 7, 9.

" VIII., Nos. 6, 7, 10.

" XV., Nos. 3, 4.

" XVI., Nos 1, 2, 3, 4, 5, 6, 7.

MISSOURI DENTAL JOURNAL:

Vol. I., Nos. 2, 3, 10.

" V., Nos. 1, 2, 3, 4, 5, 6, 7, 8, 9, 10, 11.

SOUTHERN DENTAL JOURNAL:

Vol. I., No. 3.

W. C. BARRETT.

---

### AN ITEM.

At the request of Dr. Francis, to whom I happened to mention the matter in a recent conversation, allow me to say that in dressing cases of pyorrhœa alveolaris I apply the aromatic sulphuric acid or other medicament on a fragment of Japanese bibulous-paper wrapped around a rather coarse "nerve-plugger," the point of which, for an inch or so, has been waxed. My wax is alloyed with a little resin, which improves it for this purpose, or for use on silk floss, as it is made a little more tenacious. The wax protects the steel from erosion and prevents the paper from slipping from the instrument. The instrument, thus armed, is not only excellent for medicating, but is quite effective in removing bits of dislodged concretions.

J. S. LATIMER, D. D. S.

---

THE JOURNAL OF THE BRITISH DENTAL ASSOCIATION says that Mr. Turlington, of Dublin, recently swallowed three false teeth. They stuck in the esophagus about an inch or so down, and an operation was subsequently performed with the view of removing them, but Mr. Turlington has since died.

Frequent instances are given in the medical journals of the dislodgment and swallowing of partial artificial dentures. In some cases these result in death, while in others they are removed by various means. Dentists should always caution patients wearing them to remove them at night. Neglect of this may have a fatal result.

A Sensational Case has been on trial in New York City, in which a lady brings suit against a dentist for $50,000 damages for injuries alleged to be due to mercurial poisoning from a red rubber plate. It appears that a considerable portion of one side of the face has been eaten away by some kind of an ulcer or tumor, which originated near an excoriation caused by the rubber plate, and a number of Cheap John dentists pronounced it due to "mercuric sulphide" in the plate. One dentist, who advertises sets of teeth for $6.00, who was called we suppose as an "expert," testified that "the mercury oozes out and softens the bone and the tissues of the roof of the mouth, and causes salivation, and the breaking away of the bone." The physicians generally were of the opinion that an "alveolar abscess" was the effect of the undue pressure of the plate. The defendant testified that he had for twelve years turned out on an average forty sets of teeth a week, the general price being fifteen dollars a set. The jury were unable to agree, standing eleven for the plaintiff and one for the defendant.

---

Dr. Kohn, according to the *Medical Review*, relates a case of a melancholic patient with suicidal tendencies who, in the hope of ending her life, swallowed three large spoons, each seven inches long and with a bowl about an inch and a half wide. They were all passed from the rectum, lying together, the convexity of one bowl fitting into the concavity of the other, and surrounded by a mass of fecal matter of considerable consistency. The passage of these bodies had exerted a mild peritonitis at first, and later an attack of diarrhœa, but these disturbances speedily subsided and no trouble was experienced after the spoons had been passed from the bowel. This case is almost unique, considering the large size of the spoons and the comparatively sharp edges of the bowls.

---

Sir Spencer Wells relates an early personal experience which has in it a lesson for every professional man. In the absence of the family physician, Dr. Braithwaite, he was called upon to visit a girl who was found lying unconscious in bed. Not knowing what to do he gave brandy and water as a stimulant. When Dr. Braithwaite arrived he ordered two teaspoonfuls more to be given, but when alone in the consultation with Mr. Wells, he said: "It was very wrong to give her brandy and water. This is the first stage of some eruptive fever. But a spoonful will not make any perceptible difference, and it will convince the family that I do not differ from you. If I had," he added with a smile, "perhaps they would not believe either of us."

---

John William Blake was summoned before the Sheffield (England) Police Court on the 22d ult., by the British Dental Association. He was charged with using the title "dentist" in certain advertisements on his premises, he not being registered in the Dentists' Register. For the defense it was contended that Blake was a graduate (D. D. S.) of an American Dental College, and his diploma was produced; that he had never professed to be other than a graduate of that college, and had not represented himself as a dentist registered in England. The qualification of the American Dental College is not recognized by the General Medical Council, and the court held that the defendant had infringed the act, and fined him £5, with costs.—*Dental Record.*

A LOUISVILLE MEDICAL NEWS correspondent sends that journal a communication in which those who are opposed to the revolutionary proceedings in the American Medical Association at New Orleans last year, and who decline to en dorse the present management of the Medical Congress of 1887, are branded as wide-mouthed sore-heads, monkeys, parrots, wild asses, kangaroos, and skunks, and in which the assertion is made that "this noise and din about the Congress is but the expression of disappointed ambition on the part of a few conceited orang-outangs." Truly medical men are in a nice state of feeling over the Congress.

---

DR. AUSTIN FLINT, SEN., died at his home in New York, of Cerebral Apoplexy, March 13th. Dr. Flint was one of the founders of the Medical Department of the University of Buffalo, and for some years was its Professor of the Practice of Medicine. Removing from Buffalo to New York City, he assumed the same chair in the Bellevue Hospital Medical College, which he held to the time of his death. His "Treatise upon the Practice of Medicine" has reached its seventh edition. At the time of his death he was President-elect of the International Medical Congress which proposes to hold its next meeting in America.

---

BY EXPERIMENTS made at the Bavarian Museum, a very simple and effective method of bleaching bones, to give them the appearance of ivory, has been discovered. After digesting the bones with ether or benzine to recover the fat, they are thoroughly dried and immersed in a solution of phosphoric acid and water, containing one per cent. of phosphoric anhydride. After a few hours they are removed from the solution, washed in water and dried, when they will appear as above.—*Popular Science News.*

---

A DEPARTMENT OF PHARMACY has been established in the University of Buffalo. The faculty will consist of Rudolph A. Witthaus, A. M., M. D., Professor of Pharmaceutical Chemistry. E. V. Stoddard, A. M., M. D., Professor of Materia Medica. Willis G. Gregory, M. D., Professor of Pharmacy. David S. Kellicott, Ph. D., Professor of Botany. Frank P. Vandenberg, M. D., Professor of General and Inorganic Chemistry.

---

THE JOURNAL OF THE BRITISH DENTAL ASSOCIATION for February contains a review of the transactions of the American Dental Association for 1885, in which some of the papers are severely criticised. It would be well if every member could read the notice, for the criticisms are, in the main, quite fair and disinterested. Such honest strictures may be made of infinitely greater benefit than the indiscriminate praise which tickles the ear.

---

THE AMERICAN MEDICAL ASSOCIATION will hold its thirty-seventh annual session at St. Louis, Mo., on Tuesday, Wednesday, Thursday and Friday, May 4, 5, 6 and 7, 1886. The proceedings of the last meeting at New Orleans plunged the profession of medicine into discord and contention. It is to be hoped that the coming meeting will do something toward healing the disagreements.

---

MRS. DR. T. T. MOORE, of Columbia, South Carolina, was seriously injured by being thrown from her carriage, March 19th, but is slowly recovering.

Dr. Baxter, of Toronto, Canada, says: The genial compatibility of Listerine with so many standard remedies of the materia medica gives it a very wide range of applicability in the treatment of that large class of cases benefited, relieved, and cured by the antiseptic treatment. It is the most elegant mouth wash I have ever used, and for dental use must prove invaluable.

---

The British Dental Association which was organized in 1879, has about 600 members. The American Dental Association, organized in 1859, has only about half as many. The former society is the legal organization of Great Britain, and assumes the government of the profession. The latter is purely a voluntary association, and is scientific or nothing.

---

Dr. N. S Jenkins, who for the past twenty years has been practicing dentistry in Dresden, Germany, has been decorated with the Grand Cross of the Order of Albert (an honor of the first class), by the King of Saxony.

It is a well merited tribute, and we congratulate the doctor that his worth has received such a Royal recognition.

---

Claude Bernard, the famous physiologist, needs no monument to perpetuate his fame. Yet it is gratifying to know that the great French experimentalist is duly appreciated, and that the inauguration of a statue of him took place at the College de France on the 7th of February.

---

Edward Rowan & Co., have removed their dental depot to No. 1048 Third Avenue, New York City, where they have increased facilities for supplying dentists with their specialties, as well as with standard dental goods.

---

Mr. T. Charters White is the newly elected President of the Odontological Society of Great Britain. He is well known as a histologist and microscopist. Dr. W. St. George Elliott was elected Senior Councillor.

---

Dr. J. H. Spaulding, recently of Minneapolis, Minn., has removed to Paris, where he is associated with Dr. John W. Crane in the practice of dentistry. His address is 41 Boulevard des Capucines.

---

In Preparing Anatomical Specimens for microscopical study, the tissues, if dipped in melted bayberry tallow, are firmer and can be shaved in thinner sections than where wax, or paraffine is used.

---

The Lyons Medicale reports the case of a still-born child which, by artificial respiration, was resuscitated forty-five minutes after birth, although there were previously no signs of life whatever.

---

R. I. Pearson & Co., Kansas City, have moved their dental depot to new and more commodious quarters. They will henceforth be found on the ground floor of 210 West Ninth street.

---

"What are the last teeth to come?" asked a teacher of physiology of his class. "False teeth, sir," came from the fellow at the foot.

PASTEUR did not make such an astounding discovery after all. Lots of men have been accustomed to take in the morning "a hair of the dog that had bitten him" the night before.

DR. MAGITOT, whose name is so familiar to students of dental anatomy and to the profession of America, has been elected Vice-President of the Anthropological Society of Paris.

DR. WILHELM HERBST, the originator of the Herbst method for filling teeth, will probably visit this country and attend the meeting of the American Dental Association, if it is held in San Francisco.

DR. GEORGE WATT is at times exceedingly sententious. Here is one of his epigrammatical sayings: "Only good comes from agitation. It is the pool, not the rill, that becomes offensive and putrid."

MR. J. H. BATES has purchased the advertising agency of S. M. Pettingill & Co., for forty years successfully conducted, and it will henceforth be merged in the advertising business of Mr. Bates.

FRIENDS of the National Dental Hospital and College of London have presented Oakley Coles, who has so long been connected with it, a silver inkstand and a pair of candlesticks.

A ST. LOUIS PHYSICIAN cured a case of alcoholism by prescribing opium. He then cured the opium habit by giving cocaine. He is now searching for a cure for the cocaine habit.

DR. G. C. DABOLL, formerly of Buffalo, is permanently located at No. 14 Rue de l'Opera, Paris, as may be learned by referring to his card in the advertising pages.

IT IS ANNOUNCED that the third volume of the Medical History of the War of the Rebellion is nearly completed, and will soon be issued from the press.

CONFUCIUS very wisely said : What we know, to know that we know it; what we do not know, to know that we do not know it; this is knowledge.

THE ALABAMA STATE DENTAL ASSOCIATION will meet in Montgomery, Alabama, Tuesday, April 13th, and remain in session for four days.

THE CONNECTICUT VALLEY DENTAL SOCIETY will hold its next meeting in Hartford, Conn., June 10th and 11th.

THE UNIVERSITY of Heidelberg will celebrate its 500th anniversary in August of this year.

PROF. OGDEN DOREMUS reports a second case of fatal poisoning from the local use of cocaine.

DR. GEO. L. FIELD and wife, of Detroit, have been visiting Hot Springs, Arkansas.

